# Supplementary material for: A global Youth Peacebuilding Beliefs Scale
Source: Commun Psychol. 2026 Feb 7;4:26. doi: 10.1038/s44271-025-00340-4 (PMC12886878; doi:10.1038/s44271-025-00340-4)
Supplement: Supplementary file 2 — Supplemental Material [file 44271_2025_340_MOESM2_ESM.pdf]

A Global Youth Peacebuilding Beliefs Scale

Online Supplementary Materials

Supplementary Materials

Table of Contents

Case Selection *4*

Note S1. Conflict History Overview **4**

Table S1. Summary of Key Metrics across Cases 6

Study 1 .... *7*

Sample Information **7**

Table S2. Recruitment Methods and Sample Demographics 7

Measures and Methods **7**

Note S2. Design and Procedures 7

Note S3. Sample Focus Group Questions 8

Study 2 .... *10*

Sample Information **10**

Table S4. Recruitment Methods 10

Table S5. Sample Demographic 11

Measures and Methods **12**

Note S6. Deviations from pre-registration 12

Table S7. 22-Item Youth Peacebuilding Scale 13

Table S8. Societal Responsibility, Protest Participation, and Prosocial Behaviour Scales 14

Analytic Procedure **15**

Exclusion Criteria 15

Factor Analysis 15

Exploratory Factor Analysis 15

Figure S1. *Flowchart for Factor Analysis Process* 15

Table S9. Summary of 1-6 factor models for 22-item EFA. 16

Table S10. *EFA Three-Factor Solution for 22-Item Loadings and Descriptive Statistics* 17

Table S11. *EFA Four-Factor Solution for 22-Item Loadings and Descriptive Statistics* 18

Table S12. Summary of 1-4 factor models for 18-item EFA. 19

Table S13. *EFA Three-Factor Solution for 18-Item Loadings and Descriptive Statistics* 20

Table S14. *EFA Four-Factor Solution for 18-Item Loadings and Descriptive Statistics* 21

Table S15. Summary of 1-4 factor models for 13-item EFA. 22

Table S16. *EFA Three-Factor Solution for 13-Item Loadings and Descriptive Statistics* 23

Table S17. *EFA Four-Factor Solution for 13-Item Loadings and Descriptive Statistics* 24

Reliability Testing 25

Table S18. Summary of Cronbach's Alpha and McDonald's Omega by case 25

Structural Equation Modelling 26

Table S19. Factor loadings and structural relations in Northern Ireland 26

Table S20. Factor loadings and structural relations in Colombia 27

Table S21. Factor loadings and structural relations in Israel 28

Table S22. Factor loadings and structural relations in Switzerland 29

Table S23. Comparing Model Fit with Socioeconomic Status and Political Orientation 30

Measurement Invariance 31

Table S24. Summary of Measurement Invariance Testing and Parameters Freed 31

Across-Case Comparisons ... 32
Table S25. *Across-Case Comparisons: Configural, Metric, and Scalar Invariance* ... 32
Within-Case: Northern Ireland ... 33
Table S26. *Northern Ireland: Configural, Metric, and Scalar Invariance* ... 33
Within-Case: Colombia ... 34
Table S27. *Colombia: Configural, Metric, and Scalar Invariance* ... 34
Within-Case: Israel ... 35
Table S28. *Israel Gender: Configural, Metric, and Scalar Invariance* ... 35
Within-Case: Switzerland ... 36
Table S29. *Switzerland: Configural, Metric, and Scalar Invariance* ... 36
Mean Comparisons ... 37
Table S30. Across-case mean comparisons of YPBS ... 37
Table S31. Within-case mean comparisons of YPBS: Northern Ireland ... 38
Table S32. Within-case mean comparisons of YPBS: Colombia ... 39
Table S33. Within-case mean comparisons of YPBS: Israel ... 40
Table S34. Within-case mean comparisons of YPBS: Switzerland ... 41
Descriptives ... 42
Table S35. Mean, SD, skewness, and kurtosis for all measured variables ... 42

Case Selection

Note S1. Conflict History Overview

Northern Ireland. The Northern Ireland conflict, known as "The Troubles" (1968–1998), was a sectarian and political struggle between (primarily Protestant/British) Unionists, who wished to remain part of the UK, and (primarily Catholic/Irish) Nationalists, who sought reunification with Ireland (McKittrick & McVea, 2002). Rooted in centuries of British-Irish tensions, the conflict saw violent clashes involving Nationalist and Unionist paramilitary groups (Guelke, 1999), as well as British forces. An estimated 3,600 people were killed, with civilians making up over half of the casualties (Smyth, 1998). The 1998 Good Friday Agreement largely ended the violence, though sectarian divisions persist, and sporadic unrest still occurs (e.g., around border issues and civil rights issues like language access). More recently, Northern Ireland has changed in demographics, with Catholics now outnumbering Protestants for the first time. This is linked to the rise of Ireland's main nationalist party, Sinn Féin (Pogatchnik, 2024), primarily supported by the Catholic community. This represents a historic shift in power away from the traditional status quo of Protestant leadership (Hughes & Fox, 2024).

Colombia. Colombia's conflict, spanning over 50 years, involved government forces, leftist guerrillas (notably the Revolutionary Armed Forces of Colombia (FARC) and the Army of National Liberation (ELN)), and right-wing paramilitaries (later forming the United Self-Defence Forces of Colombia (AUC)). Rooted in political and social inequality dating back to colonial times, violence escalated during the 1960s (known as *La Violencia*; Centro Nacional de Memoria Histórica, 2016). Drug trafficking later intensified the conflict, sustaining violence among and leading to mass displacement, kidnappings, and atrocities on all sides (Angelo, 2017; McDermott, 2004; Fitzpatrick & Norby, 2013), resulting in an estimated 220,000 deaths and over 5 million displaced (Centro Nacional de Memoria Histórica, 2016). The 2016 peace accord with FARC marked a major step toward resolution, though implementation challenges persist. Armed groups, including dissident factions and drug traffickers, continue to operate, contributing to violence in rural areas despite improvements.

Israel. The Israeli-Palestinian conflict is a century-old struggle over land, identity, and sovereignty constituting one of the most prominent examples of an intractable conflict (Bar-Tal 1998, 2007). Characterised by prolonged and high level violence, the conflict has been perceived by both sides as an insolvable zero-sum game (Kriesberg, 1998). Multiple peace agreements have been attempted, including the notable 1993 Oslo Accords that offered hope for a two-state solution, but peace efforts have faltered. Most recently, Hamas attacks on October 7, 2023, and the subsequent war in Gaza has further escalated the intensity of conflict with an estimate of more than 47,000 deaths and 2 million displaced, drawing unprecedented international attention to unresolved issues of statehood, security concerns, and regional power struggles.

Switzerland. Unlike the other cases listed, Switzerland has avoided major internal or external conflicts for centuries. Switzerland's policy of neutrality, established after the Congress of Vienna (1815) and solidified in both World Wars, has helped maintain peace. The country hosts numerous international organizations, including the Red Cross and UN agencies, playing a key role in global diplomacy. Intergroup divisions are present, however, notably with geographical divides by language marked by a majority group (German-speaking) and three minority groups (French-speaking, Italian-speaking, and Romansch-speaking Swiss). Furthermore, recent concerns toward immigration have risen (Riaño & Wastl-Walter, 2006), followed by debates over integration, national identity, and policies. In our paper, Switzerland

serves as a critical comparative case, representing a stable democracy without a recent history of conflict, to examine peacebuilding in contrast to conflict-affected contexts.

Table S1. Summary of Key Metrics across Cases

	Northern Ireland	Colombia	Israel	Switzerland
Conflict timeline	1 generation since peace	Half generation since peace	Ongoing conflict	No recent conflict
Global Peace Index 2024	Ireland: Rank #2<br>UK: Rank #34	#146	#155	Rank #6
World Bank Income Classification 2024	High-income	Upper-Middle Income	High-income	High-income
2022 OECD Tertiary Education Attainment %	Ireland: 54%<br>UK: 51%	28%	51%	45%
UN Human Development Index (2022)	Ireland: 0.950<br>UK: 0.940	0.758	0.915	0.967
VDEM Liberal Democracy Index	Ireland: #2<br>UK: #19	#53	#44	#4
Mandatory military service	No	No	Yes	Yes

Study 1

Sample Information

Table S2. Recruitment Methods and Sample Demographics

	Northern Ireland	***Colombia***	***Israel***	***Switzerland***
Adolescent (ages 14-17)	*N* = 46 Youth groups near Belfast (35 girls, 10 boys, 1 non-binary)	*N* = 30 3 schools in Bogota (17 girls, 13 boys)	*N* = 19 1 school near Tel-Aviv (6 girls, 13 boys)	*N* = 11 2 schools in Embrach (4 girls, 7 boys)
Young Adult (ages 18-26)	*N* = 32 Queen's University Belfast (28 women, 4 men)	*N* = 30 Konrad Lorenz University (21 women, 9 men)	*N* = 22 The Hebrew University of Jerusalem (14 women, 8 men)	*N* = 9 University of Zurich (5 women, 1 man)

Measures and Methods

Note S2. Design and Procedures

Ethical approval for the study was obtained from [redacted] University. Informed consent was obtained from all participants prior to data collection. Focus groups were conducted in the respective language (i.e., English in Northern Ireland, Spanish in Colombia, Swiss-German in Switzerland, and Hebrew in Israel) by trained facilitators. Each session lasted approximately an hour and was conducted either in-person or online using Zoom. Participants were encouraged to share their perspectives on peacebuilding, with discussions guided by a semi-structured focus group guide.

The focus group guide was developed to explore participants' experiences and perspectives on peacebuilding efforts in their respective countries. The questions focused on getting an in-depth understanding of youth's perspectives on and experiences with peacebuilding across the three types: macrosystem, mesosystem, and microsystem. Participants were also asked to discussion other methods of peacebuilding that were not mentioned on our prepared list. In contexts of intergroup conflict, the facilitator also asked about intergroup contact. Finally, the facilitator ended with a discussion on how to engage young people in peacebuilding efforts.

The interview guide was reviewed by experts in peacebuilding research and discussed with the research team from each case to ensure cultural and contextual relevance.

Note S3. Sample Focus Group Questions

Part I: Peacebuilding Activities

For each of these activities, let's talk about:

- How much [the activity] is useful/helpful to peacebuilding in NI? Why or why not?
- Can you share an example of you, a friend, or someone you know doing this? Do you think many young people your age are, or have been, involved in attended/participated in [the activity]?
    - Tell us about how it looked or worked?
    - What was happening at the time?
    - How did other people around them respond?
    - Have you done any activities at school/University like this?
    - Were you ever involved in activities like this before, like as a child or more recently?

<u>Microsystem:</u>

- Following the news
- Doing structured volunteer work
- Raising money for charity
- Participating in discussions about social or political issues/ speaking up for equality in reality/ social media
- Reaching out to a social media influencer, online newsfeed, etc. to talk about your thoughts on an issue
- Contacting someone in the government about a social or political issue

<u>Mesosystem:</u>

- Protest, street demonstrations, or rallies
- Strikes/ stay-at-home strikes/ stay-aways
- Blocking roads
- Buying/boycotting something for political reasons
- Protest art/ street theatre

<u>Macrosystem:</u>

- Voting in elections
    a) [Adolescent-only] When you are old enough, do you plan to vote?
    b) [Young adult-only] How likely would you be to vote in the next general election?
    c) Do you think voting can address the conflict?
    d) Do you think that when young people like yourself get involved in politics, they really can change the way things are run?

Besides the examples we have talked about, are there any other kinds of activities that you do to help to build peace and promote social cohesion in NI?

- What you could *personally* do, at this point in your life, to promote peace?

- Do you and your friends ever talk about the future of peace in [your country]?

Part II: Intergroup Contact

Next, I want you to think about your interactions with members of the other group:
- How often do you socialize or play sport with people from the other group?
- What about your friends, and family, do they spend time with folks from the other group? In what situations or ways?
- How many close friends from the other group do you have?
- Would you having friends from the other group be a form of peacebuilding? Why or why not?

The government and/or local councils give money every year to cross-community projects that try to help children and young people from different groups get along in [country].
- Have you ever been to any cross-community projects outside school?
- Do you think that YOUR OWN EXPERIENCE of these activities or spaces has made you like people from the other group more, like them less, or has it made no difference?
- Have you ever been actively involved in the organization, leadership, and running of a cross-community program?

Part III: Facilitating Peacebuilding

What things would help you be more involved in peacebuilding?
- For example, what could the government do to increase youth involvement?
- Do you have any influence when it comes to any of the local decisions made around here?
- Do you feel you have any influence in decisions about what happens in [your country]?
- Do you consider [your country's] government's work for peace effective? Why / why not?
- Do you feel that you know how you can make a change within the society if you want to?

Do you think that the Internet and social media (things like Facebook, Instagram, Twitter, or TikTok) has opened up new ways to promote peace and resolve conflict? Why or why not?
- Please share some examples of these either way?
- Can you share an example of positive you saw on social media.

Study 2

Sample Information

Table S4. Recruitment Methods

	Northern Ireland	***Colombia***	***Israel***	***Switzerland***
Adolescent (ages 14-17)	11 schools in Northern Ireland	Survey Company; samples from Medellin, Bogota, Barranquilla and Cali	N/A	5 schools
Young Adult (ages 18-26)	Prolific and Queen's University Belfast, and Ulster University		Survey Company	Survey Company

Table S5. Sample Demographic

		N	Age	Gender			SES	Group Identity		
			M (SD)	Male	Female	Other	M (SD)	Protestant	Catholic	Other
**Northern Ireland**	Adolescent	339	16.5 (1.45)	58%	39%	3%	4.89 (1.52)	48%	41%	11%
	Young Adult	175	21.6 (2.80)	33%	64%	3%	5.71 (1.89)	56%	40%	4%
**Colombia**	Adolescent	406	15.2 (1.14)	51%	48%	1%	5.63 (1.57)			
	Young Adult	400	20.8 (2.10)	53%	45%	2%	5.70 (1.85)			
**Israel**	Young Adult	833	25.4 (2.05)	47%	52%	1%	N/A			
**Switzerland**	Adolescent	232	15.2 (1.17)	34%	65%	1%	N/A			
	Young Adult	386	22.1 (2.68)	31%	68%	1%	N/A			

Measures and Methods

Note S6. Deviations from pre-registration

The analyses reported in this manuscript largely followed the pre-registered plan [link]. Below, we detail deviations and additions:

- **Exploratory Factor Analysis (EFA):** The pre-registration anticipated a two-factor structure, but stated we would explore other factor structures. Our EFA analyses identified a four-factor structure instead, and thus adjustments were made to the remaining analyses using this four-factor structure. In addition to the pre-registered scree plots and eigenvalues, we also use parallel analysis to determine the number of factors.
- **Confirmatory Factor Analysis (CFA):** While only an exploratory factor analysis (EFA) was pre-registered, we conducted CFA in addition to support scale validation. CFA was not pre-registered but was added to provide further evidence of the factor structure identified through EFA.
- **Measurement Invariance:** We tested measurement invariance age groups, gender, and intergroup identity as pre-registered. We also included an additional test of measurement invariance across cases to further support the validity of the scale.
- **Internal Validity:** The pre-registration stated plans to examine internal validity by correlating items within the YPBS and their relationship to corresponding behaviours. Given the 4-factor structure of the YBPS, we report the covariances of the latent subscales, rather than on an item level as noted in the pre-registration.
- **Reliability:** We report Cronbach's alpha and McDonald's omega as additions to support scale validation.
- **Convergent and Divergent Validity:** While the pre-registration stated we would examine convergent and divergent validity using a broad set of related constructs, the final analyses conducted focused on three constructs aligned with the three-level DPM framework.
- **Latent Means:** Latent means analyses were not pre-registered; We conducted them as exploratory analyses to further understand differences across groups based on the identified factor structure.

Table S7. 22-Item Youth Peacebuilding Scale

Type	ID	Item	Rationale
Macrosystem	PeaceBeliefs_2	The younger generation has the power to create a more peaceful future	
	PeaceBeliefs_5	Conversations about peace need to focus more on the future and less on the past	
	PeaceBeliefs_6	It takes a large group of people to create change/have an impact	Lowest loading (<.30) across all solutions
	Intergroup_2	In school, we should learn about conflicts between people to promote peace	In school, we should learn about conflicts between people to promote peace
	Intergroup_3	We should care less about group labels and get to know each other as individual people	
	Intergroup_4	Learning about sectarianism/discrimination is important for peacebuilding	
	Intergroup_7	Spending time with people from the other background reduces prejudices/negative stereotypes*	
	Intergroup_8	Having friends from the other group can build peace	Having friends from the other group can build peace
	Voting_1	Voting is an important way to express your beliefs	
	Voting_2	Voting makes a difference in my country	
	Voting_3	We need more young people in politics	
Mesosystem	Protest_1	I feel inspired by protests	Qualitatively different from other protest items
	Protests_2	Protests are an effective way to build peace	
	Protests_3	Protesting about social issues (e.g., LGBTQ, cost of living) can bring people together	
	Art_1	You can make a change through art.	You can make a change through art.
	Art_2	Protest art is a way to express your opinion*	
	Art_3	Art can convey symbols of peace (e.g., white dove, peace sign)	Art can convey symbols of peace (e.g., white dove, peace sign)
Microsystem	Discussion_1	I learn new things in these discussions	I learn new things in these discussions
	Discussion_2	It [discussions] helps me see a difference perspective	
	Discussion_3	Discussions can help raise awareness about peacebuilding	
	Discussion_4	I feel personally connected to the topic of peacebuilding	Lowest loading of discussion items
	Discussion_5	It's important to stay open-minded	Lowest loading of discussion items

Note: Items that are greyed out were not included in the final scale. Lighter grey represents items that were removed from the first round of EFA, and darker grey represents items that were removed from the second round. *=Item was omitted for the Israeli sample due to lack of applicability in the cultural context.

Table S8. Societal Responsibility, Protest Participation, and Prosocial Behaviour Scales

Scale	Item
**Societal Responsibility**	
[1 = Strongly disagree,<br>2 = Disagree,<br>3 = Neither agree nor disagree,<br>4 = Agree,<br>5 = Strongly agree]	If you love Northern Ireland, you should notice its problems and work to correct them<br>I oppose some Northern Ireland policies because I care about my country and I want to improve it.<br>Being concerned about national and local issues is an important responsibility for everybody
**Protest Participation**	
[1 = Yes, recently (within the past 3 months),<br>2 = Yes, but not recently (not within the past 3 months),<br>3 = No]; Recoded as binary	Attend a demonstration/protest/march
**Prosocial Behavior**	
[1 = Never,<br>2 = Sometimes,<br>3 = About half the time,<br>4 = Most of the time,<br>5 = Always]	I help my peers<br>I show recognition of the feelings of others<br>I am concerned when other people are distressed<br>I am kind towards other people<br>I am cooperative with other people<br>I am concerned for moral issues (for example, fairness, welfare of others)<br>I offer help or comfort when other people are upset

Note. For *Societal Responsibility,* participants initially responded to 6-items adapted from the Civic Responsibility Scale (Flanagen et al., 2007) and Political Efficacy Scale (Pancer et al., 2007; adapted from the California Civic Index, Kahne et al., 2005). An EFA revealed these items mapped onto a two-factor structure, not their original subscales, differentiating local versus societal levels. Mapping onto the macrosystem of broader societal norms and culture, we selected the items from the factor relating to societal levels and labelled the measure *societal responsibility*.

Analytic Procedure

Exclusion Criteria.

Factor Analysis

Exploratory Factor Analysis

Figure S1. *Flowchart for Factor Analysis Process*

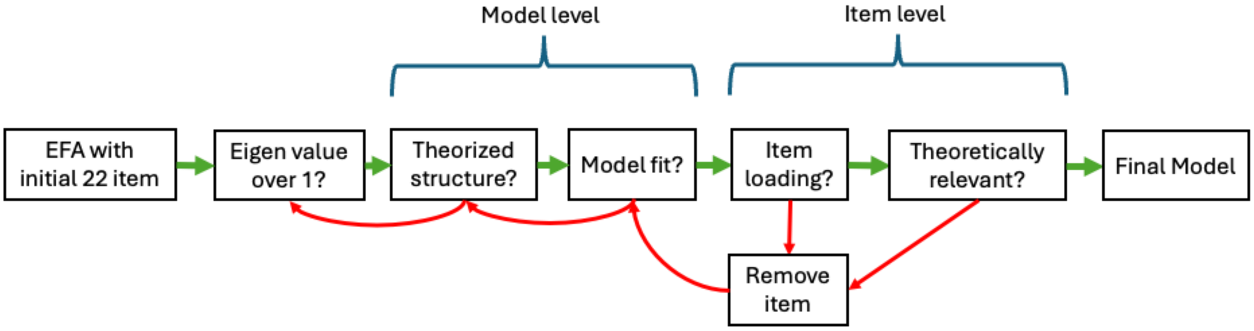

Table S9. Summary of 1-6 factor models for 22-item EFA.

Model	Variance Explained	TLI	RMSEA [90% CI]	Notes
Model #1<br>One-factor Model	Factor 1: 31%	0.695	0.104<br>[0.099, 0.11]	Not considered because low model fit
Model #2<br>Two-factor Model	Factor 1: 23.8%<br>Factor 2: 10.0%	0.747	0.095<br>[0.089, 0.101]	Not considered because low model fit
Model #3<br>Three-factor Model	Factor 1: 14%<br>Factor 2: 11.8%<br>Factor 3: 10.2%	0.820	0.08<br>[0.074, 0.086]	
Model #4<br>Four-factor Model	Factor 1: 13.7%<br>Factor 2: 11.4%<br>Factor 3: 8%<br>Factor 4: 6.3%	0.873	0.067<br>[0.06, 0.074]	
Model #5<br>Five-factor Model	Factor 1: 12.2%<br>Factor 2: 11.1%<br>Factor 3: 7.7%<br>Factor 4: 6.3%<br>Factor 5: 4.8%	0.922	0.058<br>[0.05, 0.065]	Not considered because factor variance < %5
Model #6<br>Six-factor Model	Factor 1: 11.3%<br>Factor 2: 11.1%<br>Factor 3: 7.7%<br>Factor 4: 6.4%<br>Factor 5: 5%<br>Factor 6: 2.2%	0.905	0.052<br>[0.044, 0.061]	Not considered because factor variance < %5

Note. Models not considered for item reduction are highlighted in grey.

Table S10. *EFA Three-Factor Solution for 22-Item Loadings and Descriptive Statistics*

**Items**	F1	F2	F3	Mean (SD)
Voting_1				
Voting_2				
Voting_3				
Protests_1		.64		2.84 (1.20)
Protests_2		.74		2.86 (1.17)
Protests_3		.63		3.32 (1.34)
Discussion_1	.85			3.79 (0.95)
Discussion_2	.86			3.92 (0.93)
Discussion_3	.72			3.97 (0.93)
Discussion_4	.40			3.22 (1.11)
Discussion_5	.54			4.21 (0.89)
Art_1		.62		2.72 (1.18)
Art_2		.55		3.44 (1.17)
Art_4		.44		3.67 (1.10)
Intergroup_2			.54	3.83 (1.01)
Intergroup_3			.68	4.18 (0.98)
Intergroup_4			.69	4.02 (1.00)
Intergroup_7			.65	3.88 (1.04)
Intergroup_8			.66	4.08 (0.96)
PeaceBeliefs_2			.61	4.08 (0.99)
PeaceBeliefs_5			.52	3.91 (1.00)
PeaceBeliefs_6				

Note. Factors were extracted using maximum likelihood and rotated using the oblimin method. Loadings <|.30| are not displayed.

Table S11. *EFA Four-Factor Solution for 22-Item Loadings and Descriptive Statistics*

**Items**	F1	F2	F3	F4	Mean (SD)
Voting_1					
Voting_2					
Voting_3			.34		3.89 (1.18)
Protests_1		.71			2.84 (1.20)
Protests_2		.75			2.86 (1.17)
Protests_3		.41			3.32 (1.34)
Discussion_1	.84				3.79 (0.95)
Discussion_2	.86				3.92 (0.93)
Discussion_3	.72				3.97 (0.93)
Discussion_4	.40				3.22 (1.11)
Discussion_5	.54				4.21 (0.89)
Art_1				.74	2.72 (1.18)
Art_2				.69	3.44 (1.17)
Art_4				.62	3.67 (1.10)
Intergroup_2			.55		3.83 (1.01)
Intergroup_3			.66		4.18 (0.98)
Intergroup_4			.70		4.02 (1.00)
Intergroup_7			.65		3.88 (1.04)
Intergroup_8			.64		4.08 (0.96)
PeaceBeliefs_2			.62		4.08 (0.99)
PeaceBeliefs_5			.45		3.91 (1.00)
PeaceBeliefs_6					

Note. Factors were extracted using maximum likelihood and rotated using the oblimin method. Loadings <|.30| are not displayed.

Table S12. Summary of 1-4 factor models for 18-item EFA.

Model	Variance Explained	TLI	RMSEA [90% CI]	Notes
Model #1<br>One-factor Model	Factor 1: 33%	0.699	0.115<br>[0.110, 0.112]	Not considered because low model fit
Model #2<br>Two-factor Model	Factor 1: 28%<br>Factor 2: 11%	0.768	0.101<br>[0.085, 0.108]	Not considered because low model fit
Model #3<br>Three-factor Model	Factor 1: 20%<br>Factor 2: 13%<br>Factor 3: 12%	0.863	0.078<br>[0.071, 0.085]	
Model #4<br>Four-factor Model	Factor 1: 16%<br>Factor 2: 12%<br>Factor 3: 11%<br>Factor 4: 8%	0.907	0.064<br>[0.056, 0.072]	

Note. Models not considered for item reduction are highlighted in grey.

Table S13. *EFA Three-Factor Solution for 18-Item Loadings and Descriptive Statistics*

**Items**	F1	F2	F3	Mean (SD)
Intergroup_2			.61	3.83 (1.01)
Intergroup_3			.64	4.18 (0.98)
Intergroup_4			.75	4.02 (1.00)
Intergroup_7			.69	3.88 (1.04)
Intergroup_8			.67	4.08 (0.96)
PeaceBeliefs_2			.63	4.08 (0.99)
PeaceBeliefs_5			.44	3.91 (1.00)
Voting_1			.33	3.97 (1.06)
Voting_2				
Voting_3			.39	3.89 (1.18)
Protests_2		.39		2.86 (1.17)
Protests_3		.43		3.32 (1.34)
Art_1		.79		2.72 (1.18)
Art_2		.72		3.44 (1.17)
Art_4		.58		3.67 (1.10)
Discussion_1	.83			3.79 (0.95)
Discussion_2	.88			3.92 (0.93)
Discussion_3	.68			3.97 (0.93)

Note. Factors were extracted using maximum likelihood and rotated using the oblimin method. Loadings <|.30| are not displayed.

Table S14. *EFA Four-Factor Solution for 18-Item Loadings and Descriptive Statistics*

**Items**	F1	F2	F3	F4	Mean (SD)
Intergroup_2			.49		3.83 (1.01)
Intergroup_3			.69		4.18 (0.98)
Intergroup_4			.63		4.02 (1.00)
Intergroup_7			.72		3.88 (1.04)
Intergroup_8			.69		4.08 (0.96)
PeaceBeliefs_2			.53		4.08 (0.99)
PeaceBeliefs_5			.46		3.91 (1.00)
Voting_1				.70	3.97 (1.06)
Voting_2				.49	3.65 (1.23)
Voting_3				.49	3.89 (1.18)
Protests_2		.37			2.86 (1.17)
Protests_3		.41			3.32 (1.34)
Art_1		.81			2.72 (1.18)
Art_2		.69			3.44 (1.17)
Art_4		.59			3.67 (1.10)
Discussion_1	.82				3.79 (0.95)
Discussion_2	.88				3.92 (0.93)
Discussion_3	.67				3.97 (0.93)

Note. Factors were extracted using maximum likelihood and rotated using the oblimin method. Loadings <|.30| are not displayed.

Table S15. Summary of 1-4 factor models for 13-item EFA.

Model	Variance Explained	TLI	RMSEA [90% CI]	Notes
Model #1 One-factor Model	Factor 1: 33%	0.773	0.107 [0.098, 0.116]	Not considered because low model fit
Model #2 Two-factor Model	Factor 1: 23% Factor 2: 15%	0.815	0.096 [0.087, 0.106]	Not considered because low model fit
Model #3 Three-factor Model	Factor 1: 17% Factor 2: 14% Factor 3: 13%	0.877	0.079 [0.068, 0.090]	
Model #4 Four-factor Model	Factor 1: 14% Factor 2: 11% Factor 3: 10% Factor 4: 9%	0.966	0.041 [0.26, 0.056]	

Note. Models not considered for item reduction are highlighted in grey.

Table S16. *EFA Three-Factor Solution for 13-Item Loadings and Descriptive Statistics*

**Items**	F1	F2	F3	Mean (SD)
Intergroup_3	.68			4.18 (0.98)
Intergroup_4	.60			4.02 (1.00)
Intergroup_7	.59			3.88 (1.04)
PeaceBeliefs_2	.58			4.08 (0.99)
PeaceBeliefs_5	.57			3.91 (1.00)
Voting_1				
Voting_2				
Voting_3				
Protests_2			.69	2.86 (1.17)
Protests_3			.75	3.32 (1.34)
Art_2			.44	3.44 (1.17)
Discussion_2		.72		3.92 (0.93)
Discussion_3		.88		3.97 (0.93)

Note. Factors were extracted using maximum likelihood and rotated using the oblimin method. Loadings <|.30| are not displayed.

Table S17. *EFA Four-Factor Solution for 13-Item Loadings and Descriptive Statistics*

**Items**	F1	F2	F3	F4	Mean (SD)
Intergroup_3				.73	4.18 (0.98)
Intergroup_4				.56	4.02 (1.00)
Intergroup_7				.60	3.88 (1.04)
PeaceBeliefs_2				.53	4.08 (0.99)
PeaceBeliefs_5				.54	3.91 (1.00)
Voting_1			.87		3.97 (1.06)
Voting_2			.45		3.65 (1.23)
Voting_3			.39		3.89 (1.18)
Protests_2		.58			2.86 (1.17)
Protests_3		.92			3.32 (1.34)
Art_2		.39			3.44 (1.17)
Discussion_2	.50				3.92 (0.93)
Discussion_3	1.00				3.97 (0.93)

Note. Factors were extracted using maximum likelihood and rotated using the oblimin method. Loadings <|.30| are not displayed.

Reliability Testing

Table S18. Summary of Cronbach's Alpha and McDonald's Omega by case

	Macrosystem Beliefs		Macrosystem Voting/Politics		Mesosystem		Microsystem
	α	ω	α	ω	α	ω	α
Northern Ireland	0.73	0.77	0.65	0.69	0.70	0.73	0.76
Colombia	0.68	0.76	0.79	0.80	0.67	0.70	0.81
Switzerland	0.63	0.66	0.77	0.77	0.71	0.73	0.68
Israel	0.72	0.81	0.71	0.73	0.75	-	0.73

Structural Equation Modelling

Table S19. Factor loadings and structural relations in Northern Ireland

	Std. Estimate	SE	z	*p*	*Sig.*
Latent Variables					
Macrosystem (Belief)					
Intergroup_3	0.62				
Intergroup_4	0.66	0.11	10.08	< .001	***
Intergroup_7	0.60	0.11	9.22	< .001	***
PeaceBeliefs_2	0.68	0.13	8.51	< .001	***
PeaceBeliefs_5	0.38	0.10	6.63	< .001	***
Macrosystem (Vote)					
Voting_1	0.68				
Voting_2	0.45	0.09	9.00	< .001	***
Voting_3	0.72	0.13	8.69	< .001	***
Mesosystem					
Protests_2	0.65				
Protests_3	0.83	0.12	12.21	< .001	***
Art_2	0.54	0.10	8.60	< .001	***
Microsystem					
Discussions_2	0.79				
Discussions_3	0.78	0.06	13.72	< .001	***
Regressions					
Macrosystem (Belief)					
Prosocial (Micro)	0.36	0.06	5.42	< .001	***
Protest (Meso)	-0.03	0.06	-0.53	.594	
Society (Macro)	0.34	0.05	5.03	< .001	***
Male	-0.12	0.06	-2.24	.025	*
Adult	0.08	0.06	1.76	.078	†
Protestant	-0.01	0.05	-0.14	.887	
Macrosystem (Vote)					
Prosocial (Micro)	0.18	0.08	2.59	.010	**
Protest (Meso)	0.12	0.07	2.42	.016	*
Society (Macro)	0.45	0.06	6.68	< .001	***
Male	-0.10	0.07	-1.88	.060	†
Adult	0.05	0.08	0.93	.354	
Protestant	-0.09	0.07	-1.72	.085	†
Mesosystem					
Prosocial (Micro)	0.12	0.07	2.15	.031	*
Protest (Meso)	0.13	0.08	2.66	.008	**
Society (Macro)	0.34	0.07	5.02	< .001	***
Male	-0.25	0.08	-4.94	< .001	***
Adult	-0.04	0.08	-0.82	.415	
Protestant	-0.22	0.08	-4.13	< .001	***
Microsystem					
Prosocial (Micro)	0.24	0.06	3.88	< .001	***
Protest (Meso)	-0.07	0.07	1.45	.146	
Society (Macro)	0.39	0.06	5.33	< .001	***
Male	-0.17	0.06	-3.50	< .001	***
Adult	0.11	0.07	2.16	.031	*
Protestant	-0.01	0.06	-0.30	.763	

Note. $\chi^2(113) = 217.086$, $p < .001$, RMSEA = .042 (90% CI [.031, .052]), CFI = .952, TLI = .934, SRMR = .037. $p < .001$=***, $p < .01$=**, $p < .05$ =*, $p < .10$=†.

Table S20. Factor loadings and structural relations in Colombia

	Std. Estimate	SE	z	*p*	*Sig.*
Latent Variables					
Macrosystem (Belief)					
Intergroup_3	0.54				
Intergroup_4	0.71	0.09	14.20	< .001	***
Intergroup_7	0.37	0.08	8.22	< .001	***
PeaceBeliefs_2	0.65	0.13	8.93	< .001	***
PeaceBeliefs_5	0.50	0.11	8.43	< .001	***
Macrosystem (Vote)					
Voting_1	0.78				
Voting_2	0.78	0.05	21.25	< .001	***
Voting_3	0.70	0.06	15.49	< .001	***
Mesosystem					
Protests_2	0.67				
Protests_3	0.75	0.06	19.85	< .001	***
Art_2	0.55	0.10	7.57	< .001	***
Microsystem					
Discussions_2	0.82				
Discussions_3	0.83	0.05	18.56	< .001	***
Regressions					
Macrosystem (Belief)					
Prosocial (Micro)	0.32	0.05	5.76	< .001	***
Protest (Meso)	0.04	0.06	0.99	0.321	
Society (Macro)	0.48	0.04	8.60	< .001	***
Male	0.01	0.05	0.37	0.711	
Adult	0.02	0.05	0.64	0.524	
Macrosystem (Vote)					
Prosocial (Micro)	0.21	0.08	3.85	< .001	***
Protest (Meso)	0.00	0.09	0.03	0.976	
Society (Macro)	0.35	0.05	7.34	< .001	***
Male	0.04	0.07	0.91	0.364	
Adult	0.08	0.07	1.88	0.060	†
Mesosystem					
Prosocial (Micro)	0.24	0.08	4.06	< .001	***
Protest (Meso)	0.21	0.08	5.24	< .001	***
Society (Macro)	0.26	0.05	4.97	< .001	***
Male	-0.09	0.07	-2.24	0.025	*
Adult	0.08	0.07	1.93	0.053	†
Microsystem					
Prosocial (Micro)	0.25	0.07	4.88	< .001	***
Protest (Meso)	0.10	0.08	2.95	0.003	**
Society (Macro)	0.38	0.05	7.76	< .001	***
Male	0.04	0.07	1.13	0.259	
Adult	0.06	0.07	1.50	0.136	

Note. $\chi^2(104) = 458.107$, p < .001, RMSEA = .065 (90% CI [.058, .072]), CFI = .907, TLI = .872, SRMR = .053. *p* < .001=***, *p* < .01=**, *p* < .05 =*, *p* < .10=†.

Table S21. Factor loadings and structural relations in Israel

	Std. Estimate	SE	z	*p*	*Sig.*
Latent Variables					
Macrosystem (Belief)					
Intergroup_3	0.70				
Intergroup_4	0.78	0.05	24.02	< .001	***
PeaceBeliefs_2	0.55	0.14	5.50	< .001	***
PeaceBeliefs_5	0.51	0.15	5.04	< .001	***
Macrosystem (Vote)					
Voting_1	0.64				
Voting_2	0.75	0.10	12.28	< .001	***
Voting_3	0.63	0.15	7.73	< .001	***
Mesosystem					
Protests_2	0.79				
Protests_3	0.76	0.07	14.59	< .001	***
Microsystem					
Discussions_2		0.67	0.67		
Discussions_3	0.86	0.15	9.40	< .001	***
Regressions					
Macrosystem (Belief)					
Prosocial (Micro)	0.00	0.07	0.05	0.958	
Protest (Meso)	0.12	0.08	2.83	0.005	**
Society (Macro)	0.44	0.05	9.77	< .001	***
Male	-0.10	0.07	-2.44	0.015	*
Macrosystem (Vote)					
Prosocial (Micro)	0.22	0.05	4.89	< .001	***
Protest (Meso)	-0.04	0.05	-1.09	0.278	
Society (Macro)	0.45	0.04	9.05	< .001	***
Male	-0.07	0.05	-1.76	0.079	†
Mesosystem					
Prosocial (Micro)	0.01	0.08	0.18	0.856	
Protest (Meso)	0.31	0.09	8.03	< .001	***
Society (Macro)	0.35	0.05	8.12	< .001	***
Male	-0.14	0.08	-3.76	< .001	***
Microsystem					
Prosocial (Micro)	0.22	0.06	4.60	< .001	***
Protest (Meso)	-0.02	0.06	-0.56	0.579	
Society (Macro)	0.37	0.04	7.15	< .001	***
Male	-0.13	0.05	-3.40	0.001	**

Note. $\chi^2(66) = 352.345$, p < .001, RMSEA = .074 (90% CI [.066, .083]), CFI = .898, TLI = .847, SRMR = .051. *p* < .001=***, *p* < .01=**, *p* < .05 =*, *p* < .10=†.

Table S22. Factor loadings and structural relations in Switzerland

	Std. Estimate	SE	z	*p*	*Sig.*
Latent Variables					
Macrosystem (Belief)					
Intergroup_3	0.49				
Intergroup_4	0.61	0.11	9.76	< .001	***
Intergroup_7	0.56	0.12	9.01	< .001	***
PeaceBeliefs_2	0.47	0.12	7.28	< .001	***
PeaceBeliefs_5	0.40	0.12	6.58	< .001	***
Macrosystem (Vote)					
Voting_1	0.75				
Voting_2	0.70	0.07	13.30	< .001	***
Voting_3	0.72	0.09	11.90	< .001	***
Mesosystem					
Protests_2	0.71				
Protests_3	0.76	0.07	15.52	< .001	***
Art_2	0.58	0.09	8.81	< .001	***
Microsystem					
Discussions_2	0.71				
Discussions_3	0.73	0.09	11.16	< .001	***
Regressions					
Macrosystem (Belief)					
Prosocial (Micro)	0.26	0.05	4.13	< .001	***
Protest (Meso)	0.14	0.05	3.12	.002	**
Society (Macro)	0.28	0.05	4.09	< .001	***
Male	-0.19	0.06	-3.47	.001	**
Adult	0.16	0.05	3.37	.001	**
Macrosystem (Vote)					
Prosocial (Micro)	0.19	0.07	3.36	.001	**
Protest (Meso)	0.10	0.07	2.27	.023	*
Society (Macro)	0.35	0.07	6.03	< .001	***
Male	-0.11	0.07	-2.39	.017	*
Adult	0.22	0.07	4.71	< .001	***
Mesosystem					
Prosocial (Micro)	0.16	0.07	3.07	.002	**
Protest (Meso)	0.34	0.09	2.47	< .001	***
Society (Macro)	0.21	0.07	4.25	.001	**
Male	-0.11	0.09	-1.77	.024	*
Adult	-0.16	0.07	0.37	.001	***
Microsystem					
Prosocial (Micro)	0.24	0.06	3.88	< .001	***
Protest (Meso)	0.12	0.07	2.48	.014	*
Society (Macro)	0.30	0.07	4.25	< .001	***
Male	-0.10	0.07	-1.78	.076	†
Adult	0.02	0.06	0.37	.711	

Note. $\chi^2(104) = 206.947$, p < .001, RMSEA = .040 (90% CI [.029, .051]), CFI = .957, TLI = .941, SRMR = .035. $p < .001$=***, $p < .01$=**, $p < .05$ =*, $p < .10$=†.

Table S23. Comparing Model Fit with Socioeconomic Status and Political Orientation

Model	χ^2	df	$\Delta\chi^2$	Δdf	Δp	RMSEA	CFI
Northern Ireland	216.88	113				0.042	0.952
Model with SES	227.18	122	10.238	9	.332	0.040	0.952
Colombia	461.77	104				0.065	0.906
Model with SES	479.61	113	16.018	9	.067	0.064	0.903
Model with political orientation	476.60	113	13.872	9	.127	0.063	0.905
Israel	352.34	66		8	.026	0.074	0.898
Model with SES	365.73	73	13.235	7	.067	0.072	0.896
Model with political orientation	369.68	73	14.482	7	.043*	0.072	0.898

Note. The sample from Switzerland is omitted in this table because we do not have socioeconomic status or political orientation from the participants.

Measurement Invariance

Table S24. *Summary of Measurement Invariance Testing and Parameters Freed*

Sample & Group	Configural	Metric (loadings)	Scalar (loadings + intercept)
Across-case analyses			
Northern Ireland vs. Colombia	Full	MACROBELIEF =~ Belief_3<br>MESO =~ Meso_1<br>MESO =~ Meso_2	Belief_3 ~ 1<br>Belief _2 ~ 1<br>Belief _1 ~ 1<br>Voting_2 ~ 1<br>Meso_2 ~ 1
Northern Ireland vs. Israel	Full	MACROVOTE =~ Voting_2<br>MACROBELIEF =~ Belief_4<br>MACROBELIEF =~ Belief_2<br>MACROVOTE =~ Voting_1<br>MACROBELIEF =~ Belief_1<br>MESO =~ Meso_1	Not achievable
Northern Ireland vs. Switzerland	Full	Full	Voting _2 ~ 1<br>Micro_1 ~ 1<br>Micro_2 ~ 1<br>Belief_2 ~ 1<br>Belief_1 ~ 1<br>Voting_3 ~ 1
Northern Ireland (within-case subgroup analyses)			
Age Group	Full	Full	Voting_3 ~ 1<br>Meso_2 ~ 1
Gender	Full	Full	Full
Intergroup Identity	Full	Full	Full
Colombia (within-case subgroup analyses)			
Age Group	Full	Full	Voting_2 ~ 1
Gender	Full	Full	Meso_1 ~ 1
Israel (within-case subgroup analyses)			
Gender	Full	Full	Belief_4 ~ 1<br>Belief_5 ~ 1<br>Voting_1 ~ 1
Switzerland			
Age Group	Full	Full	Full
Gender	Full	Full	Voting_3 ~ 1<br>Meso_1 ~ 1<br>Meso_3 ~ 1

Note. Parameters Freed to Achieve Partial Invariance are detailed. Latent variables are indicated by upper case (i.e., MACROBELIEF, MACROVOTE, MESO, and MICRO). Labels referencing individual item numbers can be found in Table 1 of the main manuscript.

Across-Case Comparisons

Table S25. *Across-Case Comparisons: Configural, Metric, and Scalar Invariance*

Model	χ^2	df	$\Delta\chi^2$	Δdf	Δp	RMSEA	CFI
Northern Ireland vs. Colombia							
Configural invariance	486.12	118				0.074	0.931
Metric invariance	522.87	127	36.75	9	<.001	0.072	0.929
Partial metric invariance	497.44	124	11.47	6	.079	0.070	0.923
Scalar invariance	763.28	133	285.91	9	<.001	0.087	0.871
Partial scalar invariance	501.29	128	3.85	4	.427	0.068	0.923
Northern Ireland vs. Israel							
Configural invariance	368.69	76				0.080	0.917
Metric invariance	411.33	83	42.64	7	<.001	0.079	0.917
Partial metric invariance	370.04	77	1.344	1	.25	0.080	0.917
Scalar invariance	505.04	84	135.01	7	<.001	0.091	0.881
Partial scalar invariance	-	-	-	-	-	-	-
Northern Ireland vs. Switzerland							
Configural invariance	259.03	118				0.051	0.956
Metric invariance	275.46	127	16.427	9	.058	0.050	0.954
Scalar invariance	401.55	136	126.09	9	<.001	0.064	0.919
Partial scalar invariance	285.68	130	4.53	3	.21	0.049	0.954

Within-Case: Northern Ireland

Table S26. *Northern Ireland: Configural, Metric, and Scalar Invariance*

Model	χ^2	df	$\Delta\chi^2$	Δdf	Δp	RMSEA	CFI
Age Invariance							
Configural invariance	205.18	118				0.056	0.946
Metric invariance	218.16	127	12.974	9	.164	0.055	0.943
Scalar invariance	251.24	136	34.463	9	<.001	0.060	0.928
Partial scalar invariance	229.72	134	11.56	7	.116	0.055	0.941
Gender Invariance							
Configural invariance	206.40	118				0.058	0.937
Metric invariance	214.45	127	8.0587	9	.528	0.056	0.938
Scalar invariance	250.09	136	12.741	9	.175	0.055	0.935
Intergroup Identity Invariance							
Configural invariance	215.13	118				0.062	0.934
Metric invariance	224.20	127	9.067	9	.431	0.060	0.934
Scalar invariance	236.28	136	10.481	6	.201	0.058	0.933

Within-Case: Colombia

Table S27. *Colombia: Configural, Metric, and Scalar Invariance*

Model	χ^2	df	$\Delta\chi^2$	Δdf	Δp	RMSEA	CFI
Age Invariance							
Configural invariance	394.677	118				0.078	0.914
Metric invariance	410.368	127	15.69	9	.074	0.076	0.912
Scalar invariance	432.394	136	22.026	9	.009	0.075	0.908
Partial scalar invariance	419.557	135	9.19	8	.327	0.074	0.911
Gender Invariance							
Configural invariance	393.436	118				0.079	0.912
Metric invariance	403.400	127	9.964	9	.353	0.076	0.912
Scalar invariance	428.009	136	24.609	9	.003	0.076	0.907
Partial scalar invariance	406.537	135	3.314	8	.926	0.073	0.912

Within-Case: Israel

Table S28. *Israel Gender: Configural, Metric, and Scalar Invariance*

Model	χ^2	df	$\Delta\chi^2$	Δdf	Δp	RMSEA	CFI
Configural invariance	299.480	76				0.089	0.895
Metric invariance	310.673	83	11.193	7	.130	0.086	0.893
Scalar invariance	345.734	90	35.061	7	<.001	0.088	0.880
Partial scalar invariance	315.207	87	4.534	4	.339	0.084	0.893

Within-Case: Switzerland

Table S29. *Switzerland: Configural, Metric, and Scalar Invariance*

Model	χ^2	df	$\Delta\chi^2$	Δdf	Δp	RMSEA	CFI
Age Invariance							
Configural invariance	235.364	118				0.070	0.916
Metric invariance	241.547	127	6.183	9	.722	0.067	0.917
Scalar invariance	253.753	136	12.205	9	.202	0.065	0.915
Gender Invariance							
Configural invariance	168.780	118				0.043	0.965
Metric invariance	177.066	127	7.580	9	.578	0.041	0.965
Scalar invariance	204.024	136	26.957	9	.001	0.047	0.952
Partial scalar invariance	188.788	133	11.722	6	.068	0.042	0.961

Mean Comparisons

Table S30. Across-case mean comparisons of YPBS

	Std. Estimate [95% CI]	SE	z	*p*	*Sig.*
Northern Ireland vs. Colombia					
Macrosystem (Beliefs)	-0.69 [-1.05, -0.65]	0.10	-8.32	< .001	***
Macrosystem (Voting)	-0.58 [-0.91, -0.57]	0.09	-8.55	< .001	***
Mesosystem	0.00 [-0.15, 0.15]	0.07	0.06	.954	
Microsystem	-0.47 [-0.81, -0.49]	0.08	-8.10	< .001	***
Northern Ireland vs. Israel					
Macrosystem (Beliefs)	-0.00 [-0.07, 0.07]	0.04	-0.03	.975	
Macrosystem (Voting)	0.37 [0.18, 0.55]	0.10	3.86	< .001	***
Mesosystem	-0.15 [-0.30, -0.02]	0.07	-2.23	.026	*
Microsystem	-0.27 [-0.49, -0.19]	0.08	-4.45	< .001	***
Northern Ireland vs. Switzerland					
Macrosystem (Beliefs)	-0.87 [-0.96, -0.61]	0.09	-8.80	< .001	***
Macrosystem (Voting)	-0.15[-0.34, 0.02]	0.09	-1.74	.082	†
Mesosystem	0.07[-0.07, 1.20]	0.07	0.90	.368	
Microsystem	-0.00[-0.05, 0.05]	0.02	-0.01	.993	

Table S31. Within-case mean comparisons of YPBS: Northern Ireland

	Std. Estimate	SE	z	*p*	*Sig.*
Northern Ireland Age					
Macrosystem (Beliefs)	0.64 [0.37, 0.80]	0.11	5.30	< .001	***
Macrosystem (Voting)	0.31 [0.08, 0.61]	0.14	2.57	.010	**
Mesosystem	0.40 [0.17, 0.61]	0.11	3.52	< .001	***
Microsystem	0.69 [0.39, 0.80]	0.11	5.56	< .001	***
Northern Ireland Gender					
Macrosystem (Beliefs)	-0.48 [-0.96, -0.40]	0.14	-4.77	< .001	***
Macrosystem (Voting)	-0.39 [-0.66, -0.18]	0.12	-3.47	.001	**
Mesosystem	-0.55 [-0.92, -0.43]	0.13	-5.37	< .001	***
Microsystem	-0.58 [-0.95, -0.45]	0.13	-5.46	< .001	***
Northern Ireland Intergroup Iden.					
Macrosystem (Beliefs)	-0.13 [-0.32, 0.09]	0.11	-1.1	0.279	
Macrosystem (Voting)	-0.02 [-0.25, 0.21]	0.12	-0.17	.867	
Mesosystem	0.29 [0.05, 0.47]	0.11	2.43	.015	*
Microsystem	-0.12 [-0.34, 0.10]	0.11	-1.04	.297	

Table S32. Within-case mean comparisons of YPBS: Colombia

	Std. Estimate	SE	z	*p*	*Sig.*
Colombia Age					
Macrosystem (Beliefs)	0.07 [-0.09, 0.26]	0.09	0.94	.345	
Macrosystem (Voting)	0.25 [0.10, 0.45]	0.09	3.08	.002	**
Mesosystem	0.24 [0.10, 0.47]	0.09	3.00	.003	**
Microsystem	0.17 [0.02, 0.33]	0.08	2.15	.032	*
Colombia Gender					
Macrosystem (Beliefs)	0.08 [-0.08, 0.27]	0.09	1.03	.303	
Macrosystem (Voting)	0.16 [0.01, 0.34]	0.08	2.03	.042	*
Mesosystem	0.18 [0.01, 0.42]	0.10	2.08	.038	*
Microsystem	0.16 [0.01, 0.33]	0.08	2.08	.038	*

Table S33. Within-case mean comparisons of YPBS: Israel

	Std. Estimate	SE	z	*p*	*Sig.*
Israel Gender					
Macrosystem (Beliefs)	-0.19 [-0.34, -0.02]	0.08	-2.17	.030	*
Macrosystem (Voting)	-0.34 [-0.58, -0.20]	0.10	-4.02	< .001	***
Mesosystem	-0.33 [-0.51, -0.17]	0.09	-3.93	< .001	***
Microsystem	-0.36 [-0.59, -0.23]	0.09	-4.55	< .001	***

Table S34. *Within-case mean comparisons of YPBS: Switzerland*

	Std. Estimate	SE	z	*p*	*Sig.*
Switzerland Age					
Macrosystem (Beliefs)	0.63 [0.40, 0.86]	0.12	5.30	< .001	***
Macrosystem (Voting)	0.78 [0.54, 1.01]	0.12	6.48	< .001	***
Mesosystem	-0.05 [-0.27, 0.16]	0.11	-0.53	.600	
Microsystem	0.32 [0.10, 0.55]	0.11	2.81	< .001	***
Switzerland Gender					
Macrosystem (Beliefs)	-0.48 [-0.93, -0.36]	0.14	-4.52	< .001	***
Macrosystem (Voting)	-0.21 [-0.55, -0.01]	0.14	-2.01	.044	*
Mesosystem	-0.40 [-0.68, -0.13]	0.14	-3.58	< .001	***
Microsystem	-0.29 [-0.69, -0.12]	0.14	-2.82	.005	**

Descriptives

Table S35. Mean, SD, skewness, and kurtosis for all measured variables

	Mean	SD	Skew	Kurtosis
Societal Responsibility				
Item 1	3.82	0.96	-0.68	0.26
Item 2	3.67	1.04	-0.5	-0.26
Item 3	3.66	1.02	-0.61	0.04
Protest Participation				
Item 1	0.27	0.44	1.04	-0.92
Prosocial Behaviour				
Item 1	3.78	0.93	-0.66	0.17
Item 2	3.88	0.95	-0.73	0.07
Item 3	3.73	0.99	-0.57	-0.19
Item 4	4.03	0.9	-0.88	0.6
Item 5	3.83	0.9	-0.58	0.04
Item 6	3.62	1.03	-0.44	-0.4
Item 7	3.79	0.97	-0.56	-0.24
Macrosystem (Beliefs)				
Item 1	3.56	1.2	-0.53	-0.6
Item 2	3.79	1.13	-0.83	-0.01
Item 3	3.57	1.06	-0.48	-0.18
Item 4	3.67	1.14	-0.69	-0.29
Item 5	3.71	1.14	-0.67	-0.29
Macrosystem (Voting/Politics)				
Item 1	3.97	1.1	-1.03	0.45
Item 2	3.94	1.13	-0.98	0.19
Item 3	3.92	1.13	-0.89	0.01
Mesosystem				
Item 1	2.91	1.24	-0.08	-0.99
Item 2	3.26	1.29	-0.4	-0.88
Item 3	3.59	1.12	-0.72	-0.14
Microsystem				
Item 1	3.89	0.99	-0.85	0.41
Item 2	3.84	1.01	-0.85	0.47
