# Supplementary material for: A global Youth Peacebuilding Beliefs Scale
Source: Commun Psychol. 2026 Feb 7;4:26. doi: 10.1038/s44271-025-00340-4 (PMC12886878; doi:10.1038/s44271-025-00340-4)
Supplement: Supplementary file 3 — Reporting Summary [file 44271_2025_340_MOESM3_ESM.pdf]

nature portfolio

Corresponding author(s): DBPR COMMSPSYCHOL-25-0196-T

Last updated by author(s): Jul 17, 2025

Reporting Summary

Nature Portfolio wishes to improve the reproducibility of the work that we publish. This form provides structure for consistency and transparency in reporting. For further information on Nature Portfolio policies, see our Editorial Policies and the Editorial Policy Checklist.

Statistics

For all statistical analyses, confirm that the following items are present in the figure legend, table legend, main text, or Methods section.

n/a	Confirmed	
☐	☒	The exact sample size (*n*) for each experimental group/condition, given as a discrete number and unit of measurement
☐	☒	A statement on whether measurements were taken from distinct samples or whether the same sample was measured repeatedly
☐	☒	The statistical test(s) used AND whether they are one- or two-sided<br>*Only common tests should be described solely by name; describe more complex techniques in the Methods section.*
☐	☒	A description of all covariates tested
☐	☒	A description of any assumptions or corrections, such as tests of normality and adjustment for multiple comparisons
☐	☒	A full description of the statistical parameters including central tendency (e.g. means) or other basic estimates (e.g. regression coefficient) AND variation (e.g. standard deviation) or associated estimates of uncertainty (e.g. confidence intervals)
☐	☒	For null hypothesis testing, the test statistic (e.g. *F*, *t*, *r*) with confidence intervals, effect sizes, degrees of freedom and *P* value noted<br>*Give P values as exact values whenever suitable.*
☒	☐	For Bayesian analysis, information on the choice of priors and Markov chain Monte Carlo settings
☒	☐	For hierarchical and complex designs, identification of the appropriate level for tests and full reporting of outcomes
☐	☒	Estimates of effect sizes (e.g. Cohen's *d*, Pearson's *r*), indicating how they were calculated

Our web collection on statistics for biologists contains articles on many of the points above.

Software and code

Policy information about availability of computer code

Data collection	Qualtrics
Data analysis	R, Version 2024.04.2+764 (2024.04.2+764)

For manuscripts utilizing custom algorithms or software that are central to the research but not yet described in published literature, software must be made available to editors and reviewers. We strongly encourage code deposition in a community repository (e.g. GitHub). See the Nature Portfolio guidelines for submitting code & software for further information.

Data

Policy information about availability of data

All manuscripts must include a data availability statement. This statement should provide the following information, where applicable:

- Accession codes, unique identifiers, or web links for publicly available datasets
- A description of any restrictions on data availability
- For clinical datasets or third party data, please ensure that the statement adheres to our policy

The data file and analytic codes are available at this anonymised link:
https://osf.io/mvc7k/?view_only=e930a005b46a4283a3881a928ec9c002

Research involving human participants, their data, or biological material

Policy information about studies with human participants or human data. See also policy information about sex, gender (identity/presentation), and sexual orientation and race, ethnicity and racism.

Reporting on sex and gender	Self-reported gender information was collected. We control for gender in all SEM models, and also test for measurement invariance and compare latent means by gender.
Reporting on race, ethnicity, or other socially relevant groupings	In the case of Northern Ireland, intergroup identity was coded according to participants' self-identification as either Protestant or Catholic: "In Northern Ireland, traditionally there have been two major communities: Protestant and Catholic. With which community background do you most identify?" . Participants who did not identify as one of the specified groups above were excluded from analysis. We control for intergroup identity in all SEM models for Northern Ireland, and also test for measurement invariance and compare latent means by intergroup identity in the case of Northern Ireland.
Population characteristics	Age was categorized into two groups, adolescents and young adults, based on the typical age range associated with secondary and tertiary education within each cultural context. We control for age in all SEM models, and also test for measurement invariance and compare latent means by age group.
Recruitment	Study 1:<br>- Northern Ireland: 10 youth groups near Belfast (adolescents) and 2 local universities (young adults)<br>- Colombia: 3 schools in Bogota (adolescents) and 1 local university (young adults)<br>- Israel: 1 school in Tel-Aviv (adolescents) and 1 local university (young adults)<br>- Switzerland: 2 schools in Embrach (adolescents) and 1 local university (young adults)<br>Study 2:<br>- Northern Ireland: 11 schools in Northern Ireland (adolescents), two local universities and online through Prolific (young adults)<br>- Colombia: Survey company recruiting from Medellin, Bogota, Barranquilla and Cali (adolescents and young adults)<br>- Israel: Survey company recruiting online (young adults)<br>- Switzerland: 5 schools (adolescents) and survey company recruiting online (young adults)<br><br>We recognise there is likely bias in our sample, especially for Study 1 where the sample sizes were smaller.
Ethics oversight	Ethics were approved by University College Dublin (Ireland), Konrad Lorenz University (Colombia), University of Zurich (Switzerland), and The Hebrew University of Jerusalem (Israel)

Note that full information on the approval of the study protocol must also be provided in the manuscript.

Field-specific reporting

Please select the one below that is the best fit for your research. If you are not sure, read the appropriate sections before making your selection.

☐ Life sciences ☒ Behavioural & social sciences ☐ Ecological, evolutionary & environmental sciences

For a reference copy of the document with all sections, see nature.com/documents/nr-reporting-summary-flat.pdf

Behavioural & social sciences study design

All studies must disclose on these points even when the disclosure is negative.

Study description	This is a sequential mixed-methods study for scale development.
Research sample	The sample varies across cases (see above section in "Recruitment" for more details). The sample is generally balanced in gender and age group, although some differences are observed across cases. The samples are not universally representative, although we do note that they are more representative than previous studies on similar topics.
Sampling strategy	Sampling was conducted using a mix of convenience (for youth groups and schools; less representative at the national level) and random sampling (for online recruitment through survey companies; more representative at the national level).<br><br>Quantitative (Study 1) sample size was determined using effect sizes from previous findings with G*Power and projected attrition.<br><br>Qualitative (Study 2) sample size was informed by previous work in conflict-affected societies, which reached a saturation of themes with a similar number of groups and participants.
Data collection	Qualitative Study 1: Participants completed the focus groups online (young adults) or in-person with a research assistant (adolescents).<br><br>Quantitative Study 2: Participants completed the survey independently online (young adults in Northern Ireland, Switzerland, and Israel), in-person in a supervised classroom (adolescents in Northern Ireland and Switzerland), or in-person with a data collection research assistant (adolescents and young adults in Colombia).

Timing	Study 1: Focus group data was collected between March - June in 2023 for all 4 cases.<br>Study 2:<br>- Northern Ireland: November 2023 - September 2024<br>- Colombia: February 2024 - May 2024<br>- Switzerland: January 2024 - March 2024<br>- Israel: June 2024
Data exclusions	Exclusion critera were pre-registered and based on the following: (a) born or raised in the country and (b) within the specified age ranges.
Non-participation	No participants dropped out, but they were allowed to skip any questions on the survey.
Randomization	Participants were not allocated to experimental groups.

Reporting for specific materials, systems and methods

We require information from authors about some types of materials, experimental systems and methods used in many studies. Here, indicate whether each material, system or method listed is relevant to your study. If you are not sure if a list item applies to your research, read the appropriate section before selecting a response.

Materials & experimental systems

n/a	Involved in the study
☒	☐ Antibodies
☒	☐ Eukaryotic cell lines
☒	☐ Palaeontology and archaeology
☒	☐ Animals and other organisms
☒	☐ Clinical data
☒	☐ Dual use research of concern
☒	☐ Plants

Methods

n/a	Involved in the study
☒	☐ ChIP-seq
☒	☐ Flow cytometry
☒	☐ MRI-based neuroimaging

Plants

Seed stocks	*Report on the source of all seed stocks or other plant material used. If applicable, state the seed stock centre and catalogue number. If plant specimens were collected from the field, describe the collection location, date and sampling procedures.*
Novel plant genotypes	*Describe the methods by which all novel plant genotypes were produced. This includes those generated by transgenic approaches, gene editing, chemical/radiation-based mutagenesis and hybridization. For transgenic lines, describe the transformation method, the number of independent lines analyzed and the generation upon which experiments were performed. For gene-edited lines, describe the editor used, the endogenous sequence targeted for editing, the targeting guide RNA sequence (if applicable) and how the editor was applied.*
Authentication	*Describe any authentication procedures for each seed stock used or novel genotype generated. Describe any experiments used to assess the effect of a mutation and, where applicable, how potential secondary effects (e.g. second site T-DNA insertions, mosiacism, off-target gene editing) were examined.*
